# Shigellosis Outbreak Associated with Contaminated Well Water in a Rural Elementary School: Sichuan Province, China, June 7–16, 2009

**DOI:** 10.1371/journal.pone.0047239

**Published:** 2012-10-10

**Authors:** Fan He, Ke Han, Lunguang Liu, Wei Sun, Lijie Zhang, Baoping Zhu, Huilai Ma

**Affiliations:** 1 Zhejiang Provincial Center for Disease Control and Prevention, Hangzhou, People’s Republic of China; 2 Guangdong Provincial Center for Disease Control and Prevention, Guangzhou, People’s Republic of China; 3 Sichuan Provincial Center for Disease Control and Prevention, Chengdu, People’s Republic of China; 4 Chinese Field Epidemiology Training Program, Chinese Center for Disease Control and Prevention, Beijing, People’s Republic of China; The Australian National University, Australia

## Abstract

**Objectives:**

We investigated a shigellosis outbreak in an elementary school to identify the source of infection, mode of transmission and risk factors for illness.

**Methods:**

In a case-control investigation, we compared the source of drinking water, consumption of untreated well water and suspected food items, and hygienic habits between case-students and randomly selected asymptomatic control-students, frequency-matched by class on a 1∶1 ratio.

**Results:**

18% of the 533 students and no teachers developed *Shigella*. 52%(44/85) of case-students and 17% (12/71) of control-students drank untreated well water (OR = 2.3, 95% CI = 1.1–5.8); 47% (n = 40/85) of case-students and 14% (10/71) of control-students drank untreated water from Well A (OR = 3.7, 95% CI = 1.3–11). The odds ratio increased with the amount of untreated Well A water consumed (p = 0.035, χ^2^ test for trend). Rectal swabs from 5 of 6 case-students and water from Well A yielded *Shigella flexneri* 2b.

**Conclusions:**

This shigellosis outbreak was caused by drinking untreated water from a well polluted by *Shigella flexneri* 2b.

## Introduction

Outbreaks of waterborne infection occur frequently in rural Chinese schools [1], [2]. According to data from the China Information System for Disease Control and Prevention [3], over 60 outbreaks of water-borne diseases, including hepatitis A, typhoid and cholera, were reported each year in rural Chinese schools during 2003–2009. More than 80% of these outbreaks were caused by contaminated well water.

On June 11, 2009, four patients with abdominal pain and diarrhea were admitted to a township hospital in Sichuan Province, China. The patients were all students at the same elementary school. Rectal swab samples from two of the patients were culture-positive for *Shigella*. Within three days, the number of reported cases increased to 32. We conducted an investigation to identify the source of infection, mode of transmission, and risk factors for this shigellosis outbreak. We also sought to elucidate the more general issues related to waterborne disease outbreaks in rural China.

## Materials and Methods

We conducted this outbreak investigation immediately after the outbreak was reported on June 11. This investigation was in response to an acute public health emergency event, hence was exempted from ethics approval and informed consent.

A suspected shigellosis case was defined as onset of diarrhea (≥3 times per day), vomiting, or abdominal pain during June 5–25, 2009 in a teacher or student of the elementary school or resident who lived in close proximity to the school. A probable case was defined as onset of diarrhea (≥3 times per day) plus at least one of the following symptoms: fever (≥37.5°C), vomiting, or abdominal pain. A confirmed case was a suspected or probable case plus culture confirmation of *Shigella* infection from the stool specimens or rectal swabs.

For case finding, we systematically reviewed medical records at two private clinics and the township hospital. We reviewed medical records of patients with vomiting, diarrhea, abdominal pain and fever, and applied the case definitions to identify cases. We also asked the teachers to report illness in their students during June 5–25. We also asked the teachers and staff to report their own and any family members’ illness.

In a case-control investigation, we enrolled probable or confirmed case-students in Grade 4 or above, and conducted face-to-face interviews. Students below Grade 4 could not understand the questions adequately and were excluded. Controls were randomly selected among asymptomatic students on a 1∶1 ratio, frequency matched by class. If any class had more case-students than asymptomatic students, then all asymptomatic students were selected as controls. We collected data about the onset time, symptoms and duration of illness, and history of exposure to suspected water and food items.

Rectal swabs or stool samples were collected from 97 case-students and 9 asymptomatic employees of the school cafeteria, and tested for *Vibrio cholerae, Staphylococcus aureus and Shigella*. We also collected 12 water samples (5 directly from Well A, 4 directly from Well B, 1 from a stagnant pool, 2 from the barreled water in the classrooms, and 5 from taps that drew water from Well A, 4 from taps that drew water from Well B) and tested for the same organisms. *Shigella* was also tested for in swab samples taken from the lunch boxes of 6 students, as well as from one student’s wash basin and from the surfaces in the school cafeteria. We did serologically test among *Shigella* isolates of 6 cases and 1 untreated water directly from Well A. Laboratory testing was conducted in accordance with standard practices.

Univariate analysis (Student’s t-test, Pearson chi-square test) was used to compare the age and sex distributions between the case- and control-students. Mantel-Haenszel chi-square test was used to assess risk of factors. The chi-square trend test was used to analyze dose-response relationships. Statistical analyses were performed using SPSS (version 15.0). Statistical significance was defined as P<0.05; all P-values were two-sided.

## Results

Overall, we found 118 cases (21 suspected, 33 probable, and 64 confirmed), all among the 553 students (attack rate: 21%) and none among the 51 teachers and staff of the school. Also, no cases were found among the family members of the teachers and staff or the farmers living around the school. Among 118 case-students, 105 had diarrhea and only 73(62%) had at least 3 times per day. Other common symptoms included abdominal pain (82%), fever (75%), and vomiting (23%). Many patients reported their diarrhea as initially being bloody, then turning watery.

**Figure 1:**
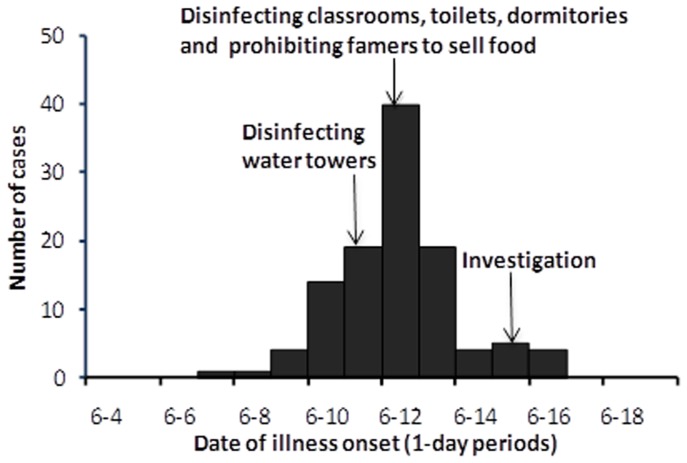
Epidemic curve. Epidemic curve for reported cases of Shigellosis by 1-day interval during a school outbreak, Sichuan Province, China, 2009.

The first case-student had onset on June 7; the number of cases rose quickly, peaking on June 12. The epidemic curve suggested a common-source exposure. After the detection of the outbreak, the local public health authority disinfected the school’s environment on June 12. Also, starting on June 11, water in the two water towers was chlorinated daily, and students were instructed not to drink water from the taps directly. Subsequently, the number of new cases declined (Figure 1).

The attack rate did not differ significantly between male and female students (P = 0.79), but did differ between boarding (27%) and commuting students (19%, P<0.05). Of the 13 classes in the school, only 7 classes were supplied with barreled water because students in these classrooms agreed to pool money to pay for the barreled water; students in the other 6 classrooms did not want to pay for the barreled water; therefore those classrooms were not supplied with barreled water. 85% of the cases occurred in classrooms not supplied with barreled water during June 10–13. The attack rate was significantly higher in classrooms not supplied with barreled water (30%) than in those supplied with barreled water (13%) (RR = 2.4, 95% CI: 1.7–3.4). Case-students in classrooms with and without barreled water both peaked on June 12, suggesting that they had shared another common source of exposure.

We enrolled 85 cases-students and 71 control-students from 10 classrooms in the case-control study. During June 2–16, 52% of case-students drank untreated water compared with 17% of control-students (OR = 2.3, 95% CI: 1.1–5.8). Drinking untreated water from Well A was a significant risk factor for developing shigellosis (OR = 3.7, 95% CI: 1.3–11); moreover, the amount of untreated water from Well A consumed was associated with the risk of shigellosis in a dose-response fashion (Table 1).

**Table 1:** Risks of shigellosis by exposure in 85 cases and 71 controls in the school, Sichuan Province, China, 2009.

Risk factors	Exposed(n)		Exposed(%)		OR	95%CI
	Cases	Controls	Cases	Controls		
Drinking water						
Untreated well water	44	12	52	17	2.3	1.1–5.8
Boiled water	40	39	47	55	1.4	0.60–3.3
Barreled purified water	42	49	49	69	1.4	0.47–4.2
Untreated water exposure						
Not drinking untreated water	39	55	46	78	Ref.	
Untreated Well A water	40	10	47	14	3.7	1.3–11
Untreated Well B water	10	8	12	11	0.40	0.08–2.1
Frequency of drinking untreated Well A water*						
Seldom	9	5	25	56	Ref.	
Usually	10	3	28	33	1.9	0.34–10
Always	17	1	47	11	9.4	0.95–94
Food exposures during June 8 to June 11						
Green beans	49	29	58	41	1.2	0.53–2.6
Potatoes	54	44	64	62	0.71	0.30–1.7
Stir-fried cucumber	33	27	39	38	1.7	0.73–3.8
Grass jelly	29	34	34	48	0.59	0.26–1.3
Cold noodles	43	38	51	54	1.1	0.48–2.4

NOTE.*Chi square for trend: χ^2^ = 4.4, P = 0.035.

Environmental investigation showed that, of the two wells the school owned, Well A was a shallow well (5 meters), constructed with stones, and was one meter away from a pond. Well B was a deep (30 meters), steel pipe well, drilled through the rock layer, and was 5 meters away from the pond. The sewage from the school and the surrounding farmers’ residents was discharged directly into the pond, without treatment. Water from Well A and Well B was pumped into Tower A and Tower B, respectively, and was directly piped to the school’s taps without treatment. The students reportedly drank untreated water frequently.

64 case-students were isolated Shigella positive. Laboratory serologically testing showed that, 5 case-students 1 untreated water directly from Well A yielded *Shigella flexneri* 2b. Other water and environmental samples, collected after chlorination and disinfection had taken place, tested negative for *Shigella*, *Vibrio cholera*, and *Staphylococcus aureus*.

## Discussion

Shigellosis is a highly infectious diarrheal disease [4] that can lead to explosive, common-source [5], [6], [7], [8] or prolonged propagated epidemics [9]. Studies have linked unboiled water [10], [11], contaminated food [12], and household contacts [13] with shigellosis. In this investigation, we documented a school outbreak of shigellosis caused by drinking untreated water from a contaminated well.

In China, many private wells supplying water to schools are built in close proximity to pollution sources, including toilets, septic tanks, sewer ditches, lakes and ponds being discharged of sewage. Water from these wells is often not treated before being piped into schools. Consequently, waterborne outbreaks with various pathogens, such as hepatitis A, Shigella, Salmonella, and norovirus frequently occured [14], [15], [16].

During this outbreak, a substantial portion of the students in the 7 classrooms supplied with barreled water were still infected, and the epidemic curve suggested that these students shared another common exposure source with students in the 6 classrooms not supplied with barreled water. These infections might be due to the fact that, even though those 7 classrooms were supplied with barreled water, sometimes the water would run out, and the students had to drink the water from the taps. Also, the students washed their hands and faces and cleaned lunch boxes, and utensils using the tap water. Because shigella has very low infectious dose, those modes of exposure to the tap water might have caused the infections in the 7 classrooms supplied with barreled water.

Waterborne outbreaks also occurred frequently during the first half of the 20^th^ century in Europe and North America [17]. After the implementation of community water treatment during the second half of the 20^th^ century, waterborne infections declined sharply [18]. A major advantage of community water treatment is its cost-effectiveness, i.e., a successful implementation of this strategy can prevent infections by many different pathogens, as well as chemical agents.

In China, regulations on the location, design and construction of private wells exist. These regulations require that the location and design of wells serving as a source of drinking water need to be approved by public health authorities. However, these regulations are rarely implemented due to insufficient enforcement and lack of coordination between public health and educational departments. In this outbreak, the implicated well, Well A, was not only shallow (5 m), but also very close to a contaminated pond in which school and residential sewage is discharged. This is a gross violation of those regulations.

In light of the findings from this investigation, we recommend that all schools in China should be provided with municipal tap water whenever available. Inspections of all existing wells should be conducted, especially of those serving school children, who due to their young age are especially prone to waterborne communicable diseases; improperly constructed wells should be abandoned and filled up. Also, all water provided to school children should be treated and chlorinated. Additionally, sewage, especially that surrounding schools, should be treated properly before discharge to prevent drinking water sources from being polluted [19].
